# Intraoperative glycemic control in patients undergoing Orthotopic liver transplant: a single center prospective randomized study

**DOI:** 10.1186/s12871-019-0918-0

**Published:** 2020-01-04

**Authors:** Sathish S. Kumar, Shawn J. Pelletier, Amy Shanks, Aleda Thompson, Christopher J. Sonnenday, Paul Picton

**Affiliations:** 1 Department of Anesthesiology, Michigan Medicine, 1H247 UH, 1500 East Medical Center Drive, SPC 5048, Ann Arbor, MI 48109-5048 USA; 2 University of Virginia, 1215 Lee st, Charlottesville, VA 22908 USA; 3 Department of surgery, Michigan Medicine, Ann Arbor, MI USA

## Abstract

**Background:**

Perioperative hyperglycemia is associated with poor outcomes yet evidence to guide intraoperative goals and treatment modalities during non-cardiac surgery are lacking. End-stage liver disease is associated with altered glucose homeostasis; patients undergoing liver transplantation display huge fluctuations in blood glucose (BG) and represent a population of great interest. Here, we conduct a randomized trial to compare the effects of strict versus conventional glycemic control during orthotopic liver transplant (OLT).

**Methods:**

Following approval by the Institutional Review Board of the University of Michigan Medical School and informed consent, 100 adult patients undergoing OLT were recruited. Patients were randomized to either strict (target BG 80–120 mg/dL) or conventional (target BG 180–200 mg/dL) BG control with block randomization for diabetic and nondiabetic patients. The primary outcomes measured were 1-year patient and graft survival assessed on an intention to treat basis. Graft survival is defined as death or needing re-transplant (www.unos.org). Three and 5-year patient and graft survival, infectious and biliary complications were measured as secondary outcomes. Data were examined using univariate methods and Kaplan-Meir survival analysis. A sensitivity analysis was performed to compare patients with a mean BG of ≤120 mg/dL and those > 120 mg/dL regardless of treatment group.

**Results:**

There was no statistically significant difference in patient survival between conventional and strict control respectively;1 year, 88% vs 88% (p-0.99), 3 years, 86% vs 84% (p- 0.77), 5 years, 82% vs 78. % (p-0.36). Graft survival was not different between conventional and strict control groups at 1 year, 88% vs 84% (p-0.56), 3 years 82% vs 76% (p-0.46), 5 years 78% vs 70% (p-0.362).

**Conclusion:**

There was no difference in patient or graft survival between intraoperative strict and conventional glycemic control during OLT.

**Trial registration:**

Clinical trial number and registry: www.clinicaltrials.gov NCT00780026. This trial was retrospectively registered on 10/22/2008.

## Background

Perioperative hyperglycemia is associated with poor outcomes including surgical site infections, possibly related to impaired phagocytic function, [1] and increased length of hospital stay. The evidence supporting strict control in critically ill patients was first published almost 16 years ago, [2] but the findings were not confirmed within other studies, the results of which suggest a lower risk of death with conventional control rather than strict control [3, 4]. During cardiac surgery [5] there is a significant reduction in pulmonary, renal complications and death with blood glucose (BG) < 200 mg/dL although there is no improved outcome with strict glucose control (target BG was between 80 and 100 mg/dl) [6]. Despite increasing trends towards treating high BG values in the perioperative setting the degree of control remains controversial [7].

Patients presenting for liver transplantation frequently display insulin resistance, termed hepatogenous diabetes [8, 9]. In addition, surgical stress, steroids, transfusion and the onset of gluconeogenesis after reperfusion of the new graft result in glucose instability. A retrospective study revealed better outcomes for patients undergoing orthotopic liver transplant (OLT) when the mean intraoperative BG was kept below 150 mg/dL [10] but prospective studies evaluating BG management during OLT are lacking. Here, we conducted a prospective randomized trial comparing strict versus conventional glucose control for patients undergoing OLT at a single academic medical center. We hypothesized that strict intra-operative blood glucose control improves one-year survival and decreases surgical complications, including infections, following OLT.

## Methods

### Study design and patient selection

Following approval from University of Michigan Medical School Institutional Review Board, Ann Arbor, Michigan (HUM 0016106/HUM 00139554) and written informed consent, adult patients undergoing OLT were randomized to either strict or conventional glycemic control (ClinicalTrials.gov ID: NCT00780026). Block randomization was performed by the study coordinator for diabetic and nondiabetic patients. Subjects were blinded to randomization group but not the clinicians. Post-operative glucose control was directed by the same standard sliding scale protocol used by the intensive care unit for both study groups. This clinical trial is in compliance with CONSORT (Consolidated Standards of Reporting Trials) statement [11].

Adult patients ≥18 years’ old who were undergoing liver transplantation were approached for study participation. The exclusion criteria included: Multi-organ transplant recipients, patients receiving an ABO incompatible liver, HIV infected patients, recipients of an organ from a HIV positive donor, patients who were unable to give informed consent, use of investigational drugs at the time of enrollment or within 30 days or 5 half-lives of enrollment, patients transplanted for hepatocellular carcinoma exceeding 3 nodules or with nodule diameter larger than 5 cm, and a history of malignancy of any organ system treated or untreated within the past 5 years, with the exception of localized basal cell carcinoma of the skin.

All patients received standard induction of anesthesia using fentanyl (1–2 mcg/kg), propofol (0.5–2 mg/kg), and muscle relaxation using either succinylcholine and/or cisatracurium as deemed appropriate by the anesthesiologist. Anesthesia was maintained with volatile agent. Infusions of cisatracurium for muscle relaxation and fentanyl for analgesia were continued throughout surgery. Methylprednisolone 500 mg was given as a standard immunosuppression to all patients. The use of inotropes and vasopressors were at the discretion of the anesthesiologist and in response to the patient’s hemodynamic status. Invasive hemodynamic monitoring included arterial and central venous access for all patients. Transesophageal echocardiography was placed unless contra-indicated by esophageal pathology or difficulty of placement. Pulmonary artery catheters were placed when thought necessary by the anesthesiologist.

### Intraoperative blood glucose management

A dedicated peripheral IV was used for all intravenous insulin administration and infusion tubing was flushed with 20 mls of insulin solution before the tubing was connected directly to the cannula without a carrier. Treatment algorithms were dictated by randomization. BG analysis were performed on arterial blood samples (Gem Premier 3000 ABG analyzer, Instrumentation Laboratory, USA) and repeated every 30 min.

The BG target for intervention in the strict glycemic control group was between 80 and 120 mg/dL. The trigger for treatment was a single BG > 130 or two BG > 120 when checked 30 min apart. Insulin treatment was by intravenous infusion, the rate of which was varied resultant upon glucose level and response to therapy according to the protocol (Table 1).

**Table 1:** Intraoperative glucose management protocol for standard glycemic control

Insulin Infusion Initiation		
BG (mg/dL)	Bolus Regular Insulin Units IV push	Initial Insulin Drip Rate Units/hr
< 180	0	0
181–200	0	2
201–300	3	3
> 300	4	4
Insulin Infusion titration		
BG mg/dL	Insulin Infusion (units per hour) Regular Insulin at Concentration of 1 unit/dL	
< 70	Hold infusion, give 25 ml of 50% dextrose IV bolus (1/2 vial). Re-check BG every 15 min until BG > 70 mg/dL, then every 30 min. When BG > 120 mg/dL restart infusion at 50% of last rate.	
70–79	Hold infusion: When BG > 120 mg/dL restart infusion at 50% of last rate	
80–99	Decrease infusion by 50% of last rate	
100–200	Continue current infusion rate unless BG reduced by > 50 mg/dl since last test, then decrease rate by 1unit/hour.	
201–250	Increase 1 unit/hr unless BG reduced by > 50 mg/dl since last test, then decrease rate by 1unit/hour.	
251–300	Increase by 2 units/hour unless BG reduced by > 50 mg/dl since last test, then decrease rate by 1unit/hour.	
301–400	Bolus 4 units regular insulin IV; increase infusion by 3 units/hour.	
> 400	Bolus 5 units regular insulin IV; increase infusion by 4 units/hour	

Target: 180–200 mg/dL

Trigger: One blood glucose > 200 or two blood glucoses > 180 when checked 30 min apart

Hypoglycemia protocol (If blood glucose < 70 mg/dL): Hold infusion, give 25 ml of 50% dextrose IV bolus (1/2 vial) and start 50mls/hr. 10% glucose. Re-check BG every 15 min until BG > 70 mg/dL, then every 30 min

The BG target for intervention in the conventional control group was 180-200 mg/dL. The trigger for treatment included one value > 200 or two values > 180 mg/dL when checked 30 min apart. Insulin infusion rate was titrated as per protocol. In addition to the continuous intravenous infusion, insulin by intravenous bolus was given if BG > 200 mg/dL (Table 2).

**Table 2:** Intraoperative glucose management protocol for strict glycemic control

Insulin Infusion Protocol					
Column 1†		Column 2‡		Column 3§	
Serum Glucose Level, mg/dL	Insulin Infusion Rate, U/h	Serum Glucose Level, m/dL	Insulin Infusion Rate, U/h	Serum Glucose Level, mg/dL	Insulin Infusion Rate, U/h
> 400	18	> 400	25	> 400	30
351–400	16	351–400	22	351–400	27
301–350	14	301–350	20	301–350	24
251–300	12	251–300	18	251–300	21
201–250	10	201–250	15	201–250	18
176–200	8	176–200	12	176–200	15
151–175	6	151–175	9	151–175	12
121–150	4	121–150	7	121–150	9
101–120	2	101–120	4	101–120	6
80–100	1	80–100	2	80–100	3
< 80	Off	< 80	Off	< 80	Off

^**1**^When glucose level is < 80 mg/dL, stop insulin infusion and initiate 50 mL/h of 10% dextrose infusion. Check glucose every 30 min until glucose level is ≥80 mg/dL - Discontinue 10% dextrose infusion. Resume insulin infusion, always in column 1. If glucose level is < 70 mg/dL, initiate treatment of hypoglycemia protocol. Restart insulin infusion in column 1 when glucose level ≥ 80 mg/dL

† Start in this column; restart in this column when insulin infusion has to be discontinued for glucose level < 80 mg/dL

‡ Patient has not reached glucose level range of 80–110 mg/dL within 2 h of using column 1 and glucose level has decreased by < 50 mg/dL over preceding 1 h

§ Patient had not reached glucose level range of 80–110 mg/dL within 2 h of using column 2 and glucose level has decreased by < 50 mg/dL over preceding 1 h

Target: 80–120 mg/dL

Trigger: One blood glucose > 130 or two blood glucoses > 120 when checked 30 min apart. Bolus doses should not be necessary using this protocol

Hypoglycemia protocol (If blood glucose < 70 mg/dL): Hold infusion, give 25 ml of 50% dextrose IV bolus (1/2 vial) and start 50mls/hr. 10% glucose. Re-check BG every 15 min until BG > 70 mg/dL, then every 30 min

Hypoglycemia was defined as a single BG < 70 mg/dL and was treated with 15 g of 50% dextrose. BG was evaluated to assess the response to treatment every 15 min until BG > 70 mg/dL.

### Outcomes

The primary outcomes were patient and graft survival at 1 year. Graft survival is defined by the United Network for Organ Sharing (www.unos.org) as death or needing re-transplant. Secondary outcomes included patient and graft survival at 3 and 5 years, hospital length of stay, biliary and infectious complications (30 days), cardiac, thromboembolic complications, renal failure needing dialysis, re-operation and wound dehiscence at one year.

### Statistical analysis

Basic descriptive statistics were calculated for demographics. Normality of continuous measures was assessed using Kolmogorov-Smirnov test. Pearson Chi-square or Fisher exact tests (for categorical variables) and independent two-tailed t-tests or Mann-Whitney U tests (for continuous variables) were used to assess baseline univariate clinical differences between patients who received strict glucose control and those who received conventional treatment. Survival and graft survival were analyzed using Kaplan Meier curves, with the log rank test for significance on an intention to treat basis.

Analysis was performed using SAS version 9.4 (SAS Institute, Cary, NC) and SPSS version 21.0 (IBM, Somers, NY). Computer-generated randomization was performed using STATA statistical software (StataCorp LP, College Station, Texas). A *p*-value of 0.05 was considered statistically significant.

A sensitivity analysis was performed to compare patients with a mean BG of ≤120 mg/dL and those > 120 mg/dL regardless of randomized group. This cutoff was chosen based on treatment threshold for the strict control group.

### Power analysis

Based on published data [2] the 1-year mortalities for the conventional and strict arms are predicted to be 22 and 9% respectively. A sample size of 89 has a greater than 80% power to detect a statistical difference between groups at the *p* = 0.05 level. A block randomization for diabetic and non-diabetic patients was used to ensure equal distribution of such patients between the treatment groups.

## Results

### Study populations and demographic characteristics

**Fig. 1:**
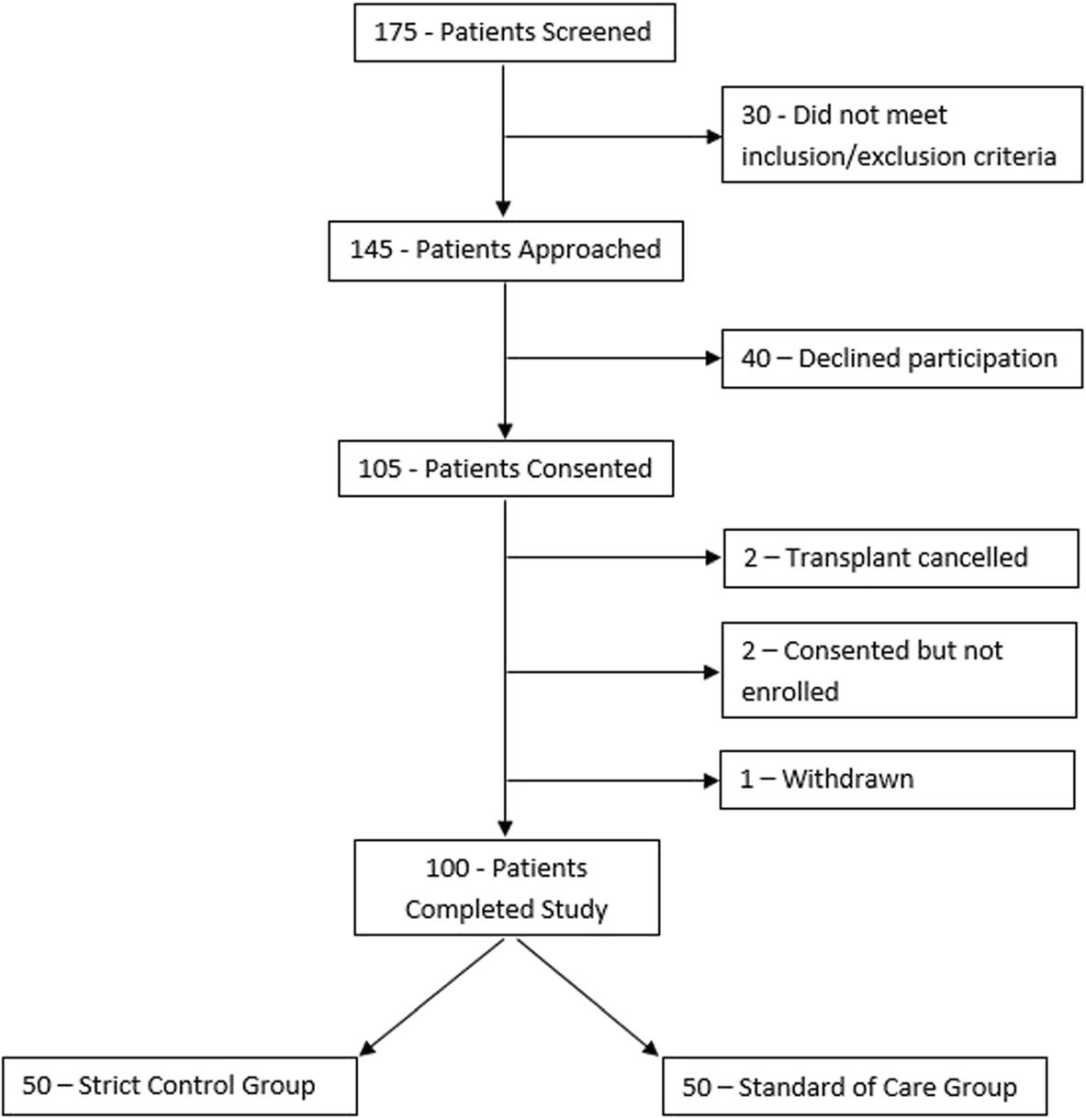
Consort flow diagram

Of the 175 patients screened, 105 patients were consented (two transplants were cancelled before enrollment, two patients were consented but not enrolled at transplant, one patient died during transplantation) and 100 patients completed the study; 50 were randomized to the conventional group and 50 were randomized to the strict glucose control group (Fig. 1). All patients were included for analysis and no patients were lost to follow up. There were no demographic or comorbidity differences between groups (Table 3). Differences in transplant characteristics and surgical factors were not demonstrable between groups (Table 4).

**Table 3:** Population and Baseline Characteristics

	Current Standard of Care (*N* = 50)	Strict Glucose Control (*N* = 50)	P-Value
Patient Demographics			
Age	55.0 [50.0 to 59.0]	54.0 [48.0 to 57.0]	0.201
*Race*			0.580
White	41 (82.0)	41 (82.0)	
African American	4 (8.0)	5 (10.0)	
Asian	1 (2.0)	0 (0.0)	
Other	4 (8.0)	4 (8.0)	
*WHO BMI Classification*			0.415
Underweight	0 (0.0)	0 (0.0)	
Normal	12 (24.5)	10 (20.0)	
Overweight	18 (36.7)	14 (28.0)	
Obese	19 (38.8)	26 (52.0)	
Female Sex	14 (28.0)	12 (24.0)	0.648
Pre-Existing Conditions			
Diabetes	16 (32.0)	13 (26.0)	0.509
CAD	4 (8.0)	4 (8.0)	0.999
MELD Score	20.5 [15.0 to 23.0]	19.5 [12.5 to 25.5]	0.744
*Cause of Liver Disease*^*1*^			
Alagille’s Syndrome	0 (0.0)	2 (4.0)	0.495
Alpha-1 Antitrypsin Deficiency	4 (8.0)	0 (0.0)	0.118
Hepatocellular Carcinoma (non-Fibrolamellar)	13 (26.0)	19 (38.0)	0.198
Alcohol-related Cirrhosis	18 (36.0)	12 (24.0)	0.190
Cryptogenic Cirrhosis	4 (8.0)	5 (10.0)	0.999
Primary Biliary Cirrhosis	2 (4.0)	1 (2.0)	0.999
Secondary Biliary Cirrhosis	1 (2.0)	0 (0.0)	0.999
Cystic Fibrosis	1 (2.0)	0 (0.0)	0.999
Hemochromatosis	1 (2.0)	0 (0.0)	0.999
Hepatitis C, Chronic	18 (36.0)	18 (36.0)	0.999
Hepatitis, Chronic Active	2 (4.0)	3 (6.0)	0.999
Hepatic Failure (Idiopathic)	1 (2.0)	0 (0.0)	0.999
HCC	1 (2.0)	1 (2.0)	0.999
Hypertensive Nephrosclerosis	1 (2.0)	0 (0.0)	0.999
Polycystic Liver Disease	1 (2.0)	0 (0.0)	0.999
Non-Alcoholic Steatohepatitis	2 (4.0)	4 (8.0)	0.678
Primary Sclerosing Cholangitis	5 (10.0)	8 (16.0)	0.554
Unknown	1 (2.0)	1 (2.0)	0.999
Wilson’s Disease	0 (0.0)	1 (2.0)	0.999

^1^Cause of liver disease is not mutually exclusive. Data are presented as frequency (%) or median [25th percentile to 75th percentile], as appropriate

**Table 4:** Transplant characteristics, surgical factors, and post-surgical complications

	Current standard of care (*N* = 50)	Strict glucose control (*N* = 50)	P-Value
Transplant Characteristics and Surgical Factors			
High Risk Donor	5 (10.0)	7 (14.0)	0.538
Warm Ischemic Time (Minutes)	26.0 [23.0 to 32.0]	27.0 [23.0 to 33.0]	0.916
Cold Ischemic Time (Minutes)	400.0 [323.0 to 488.0]	385.5 [333.0 to 429.0]	0.572
Total Ischemic Time (Minutes)	431.0 [354.0 to 522.0]	415.5 [367.0 to 455.0]	0.575
Total Surgical Time (Minutes)	313.0 [281.0 to 369.0]	329.9 [307.0 to 375.8]	0.228
Intraoperative Blood Product Usage			
Packed red cells (mL)	2450.0 [1050.0 to 3850.0]	2450.0 [1400.0 to 3500.0]	0.475
Platelets (mL)	500.0 [500.0 to 750.0]	625.0 [375.0 to 750.0]	0.667
Cryoprecipitate (mL)	200.0 [100.0 to 200.0]	200.0 [100.0 to 400.0]	0.438
Cell Saver (mL)	999.0 [500.0 to 1425.0]	848.0 [700.0 to 1600.0]	0.645

Data are presented as frequency (%) or median [25th percentile to 75th percentile], as needed

### Insulin dose, treatment response and hypoglycemia

**Fig. 2:**
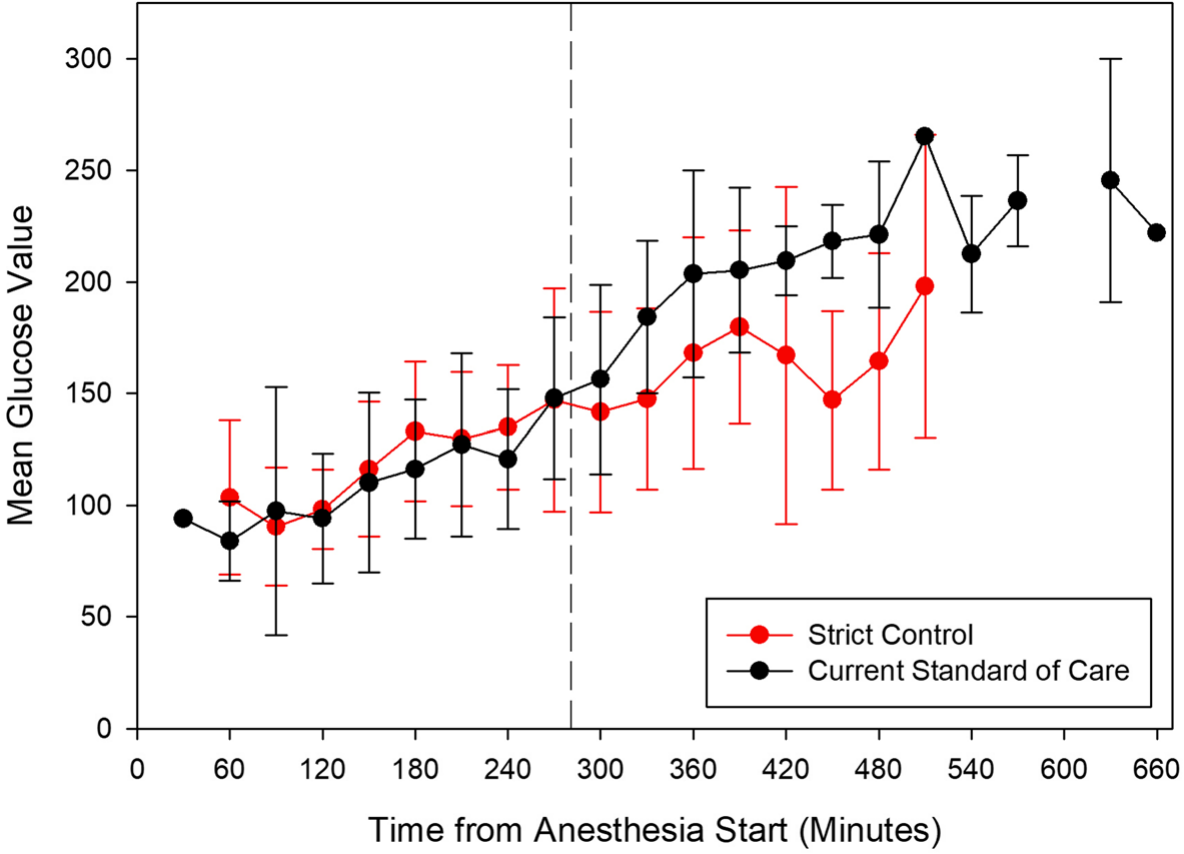
Mean intraoperative blood glucose with 95% confidence intervals for standard and strict glycemic control groups. The vertical dashed line represents the median revascularization time, which was similar in both groups

Within the conventional group the mean intraoperative BG was 143.3 mg/dL [interquartile range (IQR) 123.8 mg/dL to 167.1 mg/dL] and for the strict group 130.7 mg/dL [IQR 112.2 mg/dL to 154.8 mg/dL] (*p* = 0.020, Table 5). Patients in the strict control group received more insulin (median: 24.4 units [IQR 14.2 to 38.0] vs. 10.0 units [IQR 5.9 to 17.8], *p* < 0.001). Mean BG values over time showed statistically significant difference (*p* = 0.037) between the strict and standard control group with a divergent trajectory following reperfusion of donor graft (Fig. 2). Within the conventional group, seven patients were exposed to at least one hypoglycemic incident; six patients had one episode, one patient had two episodes. Within the strict glucose control group 13 patients exhibited one hypoglycemic episode.

**Table 5:** Glucose control

	Current standard of care (*N* = 50)	Strict glucose control (*N* = 50)	P-Value
Total No. of Hypoglycemia Incidents			0.125
0	43 (86.0)	37 (74.0)	
1	6 (12.0)	13 (26.0)	
2	1 (2.0)	0 (0.0)	
Hypoglycemia at any time	7 (14.0)	13 (26.0)	0.134
Total Insulin Dosage	10.0 [5.9 to 17.8]	24.4 [14.2 to 38.0]	< 0.001
Mean Intraoperative Glucose	143.3 [123.8 to 167.1]	130.7 [112.2 to 154.8]	0.020

Data are presented as frequency (%) or median [25th percentile to 75th percentile], as needed

### Survival and graft loss

**Fig. 3:**
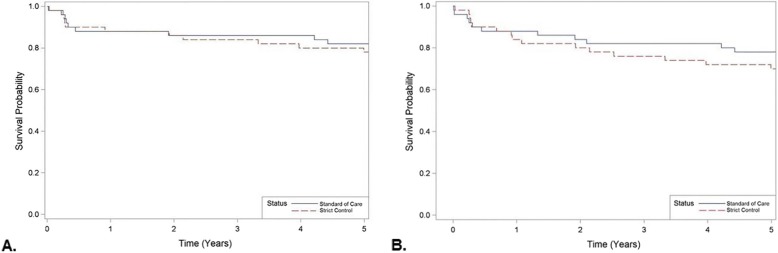
Overall survival for standard and strict glycemic control groups with time along the X-axis and survival probability along the Y-axis. A) Five-year mortality, log- rank test *p* = 0.490. B) Five-year graft survival log-rank test *p* = 0.303

The primary study (one-year survival) and secondary outcomes for graft and patient (three and five-year survival) are represented in Table 6. Patient survival was not different between conventional and strict groups respectively, (1 year: 88% vs 88%, *p* = 0.999; 3 years: 86% vs 84%, *p* = 0.779; 5 years: 82% vs 78%, *p* = 0.617). There was no statistical difference in causes of mortality between the two groups (data not shown). Graft survival was not different between conventional and strict control groups respectively, (1 year: 88% vs 84%, *p* = 0.564; 3 years: 82% vs 76%, *p* = 0.461; 5 years: 78% vs 70%, *p* = 0.362). In-hospital graft survival was not statistically significant between treatment groups (*p* = 0.118). Kaplan-Meier 5-year survival rate not reveal any statistically significant difference between the two groups (*p* = 0.490, Fig. 3a). Fig. 3b shows the same trend for the 5-year graft survival rate (*p* = 0.303). There was no difference in postoperative ICU stay between groups (median: 3.0 days [IQR 2.0 to 5.0 for both]; *p* = 0.934).

**Table 6:** Post-surgical complications

	Current standard of care (*N* = 50)	Strict glucose control (*N* = 50)	P-Value
Survival Outcomes			
Graft Loss	11 (22.0)	16 (32.0)	0.260
Death	9 (18.0)	12 (24.0)	0.461
Overall Survival			
30 Day Survival	49 (98.0)	49 (98.0)	0.999
1 Year Survival	44 (88.0)	44 (88.0)	0.999
3 Year Survival	43 (86.0)	42 (84.0)	0.779
5 Year Survival	41 (82.0)	39 (78.0)	0.617
Graft Survival			
30 Day Graft Survival	48 (96.0)	49 (98.0)	0.999
1 Year Graft Survival	44 (88.0)	42 (84.0)	0.564
3 Years Graft Survival	41 (82.0)	38 (76.0)	0.461
5 Years Graft Survival	39 (78.0)	35 (70.0)	0.362
Complications			
Bile Leak	15 (30.0)	14 (28.0)	0.826
Biliary Stricture	20 (40.0)	13 (26.0)	0.137
CVA	1 (2.0)	0 (0.0)	0.999
Hepatic Arterial Stricture	3 (6.0)	1 (2.0)	0.617
Major Cardiac Event	5 (10.0)	4 (8.0)	0.999
Portal Vein Thrombosis	3 (6.0)	3 (6.0)	0.999
Re-operation: Bleeding	6 (12.0)	10 (20.0)	0.275
Re-operation: Other	9 (18.0)	15 (30.0)	0.160
Renal Failure - Dialysis	9 (18.0)	6 (12.0)	0.401
Wound Dehiscence	5 (10.0)	12 (24.0)	0.062
Infections			
Bacterial	26 (52.0)	27 (54.0)	0.841
Fungal	7 (14.0)	11 (22.0)	0.298
Transplant Incision Wound	7 (14.0)	9 (18.0)	0.585
Viral	6 (12.0)	4 (8.0)	0.505
Hospital Outcomes			
Length of Stay (days)	11.0 [8.0 to 19.0]	12.5 [8.0 to 19.0]	0.384

Data are presented as frequency (%) or median [25th percentile to 75th percentile], as needed

### Biliary, infectious and other complications

Despite some clinically significant differences between the treatment groups, there were no statistically significant differences between the strict and conventional group for any secondary outcome measures which included bile leak (*p* = 0.826), re-operation for bleeding (*p* = 0.275), renal failure needing dialysis (*p* = 0.401), bacterial infection (*p* = 0.841), fungal infection (*p* = 0.298), wound infection (*p* = 0.585) and viral infection (*p* = 0.505) (Table 6).

### Sensitivity analysis

**Fig. 4:**
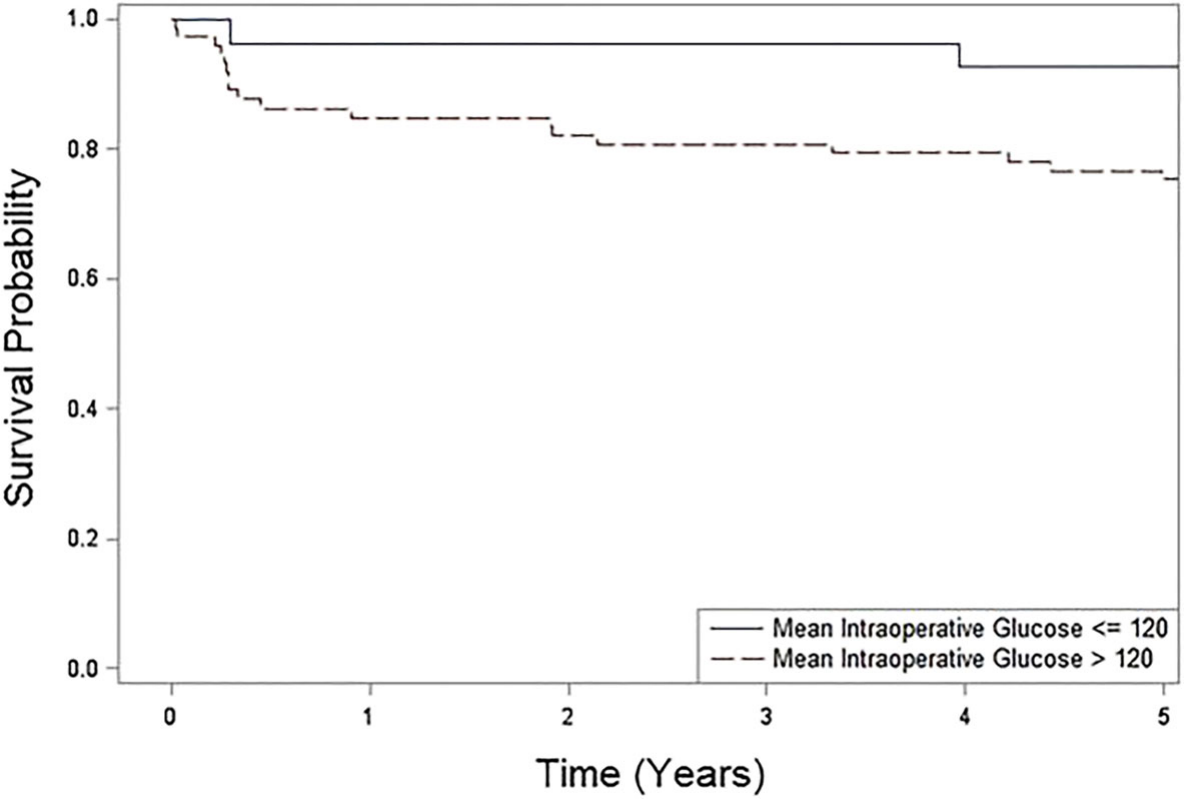
Overall 5-year patient survival comparing patients with mean blood glucose less than or equal to 120 mg/dL to those with a mean blood glucose greater than 120 mg/dL with time along X-axis and survival probability along Y-axis. The log-rank test *p* = 0.047

A sensitivity analysis was performed to compare patients with a mean BG of ≤120 mg/dL and those > 120 mg/dL, regardless of treatment group, to assess the response to insulin treatment or insulin resistance. The sensitivity analysis included all 100 patients; of those with BG < 120 mg/dL, 19 (70.4%) were from the strict control group and 8 (29.6%) were from the conventional group. There was statistically significant improved survival for patients with a mean intraoperative BG of ≤120 mg/dL with a log rank *p* value of 0.047 (Fig. 4).

After adjustment for patient comorbidities, chronic preoperative steroid use, and intraoperative transfusion using a multivariable Cox proportional hazard model, blood glucose ≤120 mg/dL did not show any significant difference in survival.

## Discussion

We report the first randomized study directly comparing strict versus conventional glucose control during the intraoperative period of liver transplantation with comprehensive long term follow up. We found no difference in patient or graft survival after OLT between the conventional and strict glucose control regimens when analyzed on an intention to treat basis. However, the sensitivity analysis revealed improved survival for those patients with a mean blood glucose ≤120 mg/dL regardless of treatment group, perhaps identifying a group of ‘responders’ or implying the requirement for more aggressive insulin management than that used within our strict control group.

Although patients in the strict group received greater than twice the dose of insulin and exhibited an approximate 10% reduction in mean intraoperative blood glucose when compared to the conventional group; these differences may not have been large enough to produce a clinically significant difference in the outcomes measured. However, most divergence in blood glucose between groups can be appreciated only after reperfusion perhaps illustrating that reliance on differences in the mean of the total operative time to be limited. The strict glucose regimen was modified from the landmark NICE-SUGAR trial, [12] the most aggressive regimen published to date, and associated with a 7% incidence of severe hypoglycemia (< 40 mg/dL). Within the conventional group, 7 patients were exposed to at least one hypoglycemic incident; 6 patients had one episode, 1 patient had two episodes. Within the strict glucose control group 13 patients exhibited 1 hypoglycemic episode. Numerically, hypoglycemic episodes were higher in the strict control group although there was no statistically significant difference between the number of patients exposed to hypoglycemia or the total number of hypoglycemic episodes between groups. The routine provision of abundant anesthesia resources for liver transplantation allowed for frequent glucose monitoring and rapid intervention for changes in plasma glucose when detected and may enhance safety for aggressive glucose management in this patient population. Patients undergoing liver transplant have the potential to exhibit hyperglycemia due to insulin resistance [9] and the exogenous administration of steroids may make hypoglycemia less likely; it appears that aggressive insulin therapy is not associated with a greater risk of hypoglycemia compared to conventional control and can be managed safely during the intraoperative phase of liver transplantation.

We found a statistically significant difference in patient survival if mean BG was less than 120 mg/dl in the sensitivity analysis although the adjusted analysis did not show any significant difference A recent prospective study investigated postoperative glycemic control and impact on outcomes at one year following liver transplantation and revealed a reduced incidence of infection with BG levels of 140 mg/dl but a higher incidence of moderate hypoglycemia (41–70 mg/dl) was described [13]. We did not continue randomization into the postoperative period but doing so as well as monitoring all elements of glucose control throughout the first postoperative year are likely of critical importance for the outcomes measured and represent a design flaw within this study.

Hyperglycemia is associated with a spectrum of poor outcomes including poor graft survival [14] and in animal studies is associated with defective handling of calcium in cardiac myocytes resulting in poor cardiac function [15]. Insulin treatment appears to reduce afterload and improve ventricular relaxation although changes in myocardial perfusion or contractility have not been demonstrated [16]. Treatment of hyperglycemia, especially in patients with non-insulin dependent DM, has been shown to improve immune function, hypercoagulability and possibly reduce the risk of infectious complications [1]; it is postulated that hyperglycemia results in impaired phagocytosis secondary to high intra-cellular calcium concentrations. In addition to hepatgeneous diabetes, immunosuppression with steroids and stress hyperglycemia likely contribute to profound fluctuations in BG levels during liver transplantation [8, 17, 18]. It is interesting to note that we found no differences in infectious complications between groups here. The precise mechanism of stress induced hyperglycemia is not clear but it is associated with poor post-operative outcomes following non-cardiac surgery [19]. Insulin resistance is likely a primary factor and general anesthesia per se may be contributory; a recent animal study reveals an increase in insulin resistance by almost 50% and insulin effects at the liver were almost completely suppressed [20]. Liver transplantation is associated with potential for significant blood loss and transfusion. Citrate phosphate dextrose (CPD) preservative solutions contribute to serum glucose; there was no difference in transfusion requirements between treatment groups.

Despite decades of research conducted within the intensive care setting glucose control remains controversial and strict control is currently not recommended for critically ill patients [21]. The thresholds to treat hyperglycemia in the cardiac surgical population may be lower compared to non-cardiac surgical patients potentially related to the effects of metabolic syndrome and hyperglycemia during cardiopulmonary bypass [22]. It is possible that patients undergoing liver transplantation share similarity with cardiac surgical patients as signaled by the results of our sensitivity analysis. There is a lack of robust data from the perioperative setting in the non-cardiac surgical patients. Our current practice is mostly based upon retrospective investigations which suggests BG > 200 mg/dL is associated with poor outcomes [23, 24]. Any improved outcome implied from our sensitivity analysis may not only impact OLT but may be of broader significance for other non-cardiac surgical patients as there is lack of good evidence in defining treatment strategies As implied by our data, it is possible to implement tight control safely with careful monitoring of BG during the intraoperative phase of an OLT [25]. Outcome differences in perioperative glycemic control may not be solely related to glycemic control per se, but may also be attributed to differences in the level of supervision, accuracy of monitoring, clinical setting and infusion practices [26].

Previous studies have reported that glucose variability was associated with acute kidney injury following OLT [24, 27, 28] which was not related directly to hyperglycemia or hypoglycemia. However, this study did not detect a difference in the requirement for dialysis post-operatively between groups but we did not measure glucose variability. In terms of detecting glucose variability, technological advances for continuous glucose monitoring and real time alerting systems may be helpful in achieving stability of BG [29, 30]. Intravenous insulin infusions have advantage over [31] subcutaneous bolus or the widely used sliding scale in maintaining steady state to prevent glucose variability [32–34].

There are limitations with our study. The sample size was small and the study population was heterogeneous. We did not achieve the target BG in the strict group, despite the use of an aggressive insulin protocol, potentially related to insulin resistance seen in patients with liver disease and the measurable difference in BG between groups may have been of insufficient magnitude to result in a meaningful clinical difference between groups. Although current evidence does not recommend strict control in the perioperative period, our strict control group was defined based on the existing literature at the time of study initiation. However, the statistically significant difference in the intraoperative BG level between groups diverged as the case progressed. The study focused exclusively on intraoperative glycemic control; including the immediate postoperative phase would have been a major improvement in study design. However, differences in patient survival following liver transplant are associated with intraoperative BG control [10] and a large retrospective study in non-cardiac surgery found that a higher mean intraoperative BG increased the odds for higher post-operative BG, [35] implying that control of the intraoperative phase of care is a rational research question. In addition, poor glycemic control in the intraoperative period adversely impacts outcome [5, 36, 37] yet the influence of postoperative management cannot be underestimated [35]. Lastly, we did not evaluate glucose homeostasis in donors but improved recipient outcome has been achieved with improved glycemic control in donors [38]. A direct relationship between donor and recipient glucose variability may exist and be an unmeasured confounder within this study.

## Conclusion

In conclusion, there is no patient or graft survival advantage with strict glucose management compared to conventional glucose management during the intraoperative phase for patients undergoing liver transplantation when evaluated on an intention to treat basis.

## Data Availability

The datasets used and/or analysed during the current study are available only after de-identification from the corresponding author on reasonable request.

## Abbreviations

BG: Blood glucose
OLT: Orthotopic liver transplant
